# Ecology and Physiology of the Pathogenic Cyanobacterium *Roseofilum reptotaenium*

**DOI:** 10.3390/life4040968

**Published:** 2014-12-15

**Authors:** Laurie L. Richardson, Dina Stanić, Amanda May, Abigael Brownell, Miroslav Gantar, Shawn R. Campagna

**Affiliations:** 1 Department of Biological Sciences, Florida International University, Miami, FL 33199, USA; E-Mails: dina.stanic@gmail.com (D.S.); Abigael.Brownell@algenol.com (A.B.); gantarm@fiu.edu (M.G.); 2 Department of Chemistry, University of Tennessee, Knoxville, TN 37996, USA; E-Mails: Amanda.May@vanderbilt.edu (A.M.); campagna@ion.chem.utk.edu (S.R.C.)

**Keywords:** cyanobacteria, black band disease, pathogen, polymicrobial disease, *Roseofilum reptotaenium*, metabolomics

## Abstract

*Roseofilum reptotaenium* is a gliding, filamentous, phycoerythrin-rich cyanobacterium that has been found only in the horizontally migrating, pathogenic microbial mat, black band disease (BBD) on Caribbean corals. *R. reptotaenium* dominates the BBD mat in terms of biomass and motility, and the filaments form the mat fabric. This cyanobacterium produces the cyanotoxin microcystin, predominately MC-LR, and can tolerate high levels of sulfide produced by sulfate reducing bacteria (SRB) that are also associated with BBD. Laboratory cultures of *R. reptotaenium* infect coral fragments, suggesting that the cyanobacterium is the primary pathogen of BBD, but since this species cannot grow axenically and Koch’s Postulates cannot be fulfilled, it cannot be proposed as a primary pathogen. However, *R. reptotaenium* does play several major pathogenic roles in this polymicrobial disease. Here, we provide an overview of the ecology of this coral pathogen and present new information on *R. reptotaenium* ecophysiology, including roles in the infection process, chemotactic and other motility responses, and the effect of pH on growth and motility. Additionally, we show, using metabolomics, that exposure of the BBD microbial community to the cyanotoxin MC-LR affects community metabolite profiles, in particular those associated with nucleic acid biosynthesis.

## 1. Introduction

### 1.1. Cyanobacteria and Black Band Disease of Corals

The role of cyanobacteria as harmful microorganisms has for the most part been that of cyanotoxin producers that degrade water quality via extracellular accumulation of toxins, which then affect animals or humans that consume the water [1,2]. To our knowledge, no cyanobacterium has been shown to serve as a direct pathogen. One cyanobacterial species, however, does appear to play an important role in a polymicrobial disease that infects and can kill a eukaryotic host. This is the predominant cyanobacterium associated with black band disease (BBD) of corals.

**Figure 1:**
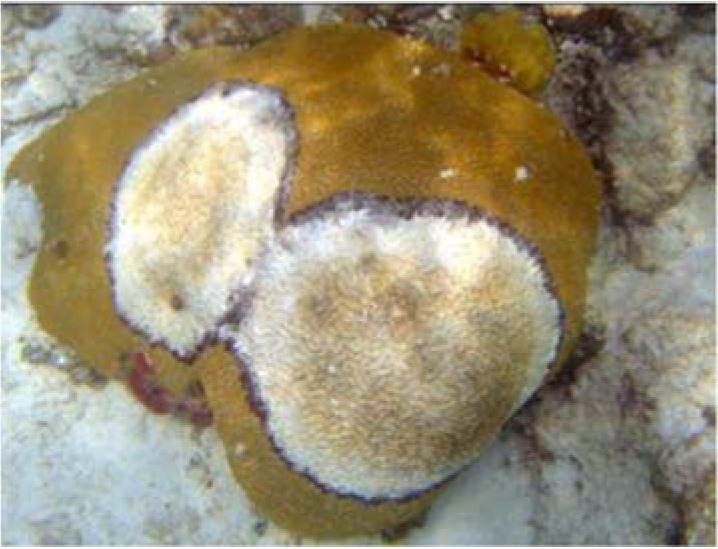
Black band disease on *Diploria strigosa*, St. John, US Virgin Islands. Two initial infections resulted in two disease rings. Image by C. Rogers.

BBD was first observed on scleractinian (stony) corals in the Caribbean in the 1970s [3]. It has since spread worldwide and is now globally distributed on tropical and sub-tropical reefs throughout the Pacific and Atlantic Oceans and the Red Sea [4] and has expanded its range throughout the wider Caribbean [5]. It was reported to infect 19 species of scleractinian and six species of gorgonian (soft) corals) on Caribbean reefs [4]; since this report the host species range has increased (L. Richardson, personal observation). BBD appears as a dense black band, several mm to several cm wide but only *ca.* 1 mm thick, that moves across a living coral colony completely lysing tissue as it progresses (Figure 1). The band often initiates from a central point on the top or side of the coral colony and migrates as an expanding ring. It is a seasonal disease, appearing when water temperatures rise above 29–30 °C and disappearing when the temperature cools below 27.5 °C [6]. It is highly infective and can be easily transmitted to healthy corals either by direct inoculation using the BBD mat in field and laboratory manipulations [7], through the water column [7], or by butterfly fish acting as a vector [8]. Often the BBD mat has a very obvious population of filamentous sulfide oxidizers that carry out a vertical migration within the horizontally migrating mat [9].

**Figure 2:**
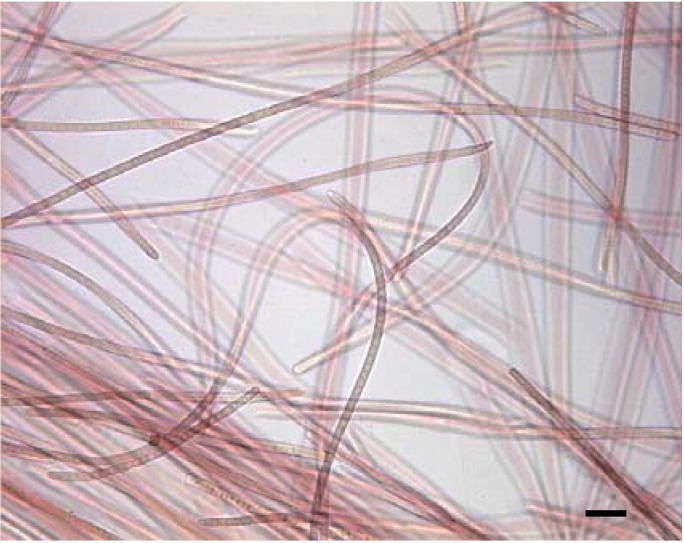
Laboratory culture of *Roseofilum reptotaenium* strain 101-1 isolated from black band disease. Note the presence of one tapered and one rounded end of the filaments. The red pigment is phycoerythrin [18]. Scale bar = 10 μm. Image by A. Brownell.

Before the widespread use of genetic analysis for taxonomic identification of organisms, BBD cyanobacteria were classified into the genera *Oscillatoria* and *Phormidium* based on morphology [10,11]. With the advent of sequencing of the 16S rRNA gene, several research groups [12,13,14,15] detected a dominant cyanobacterial ribotype present in BBD on reefs world-wide. This ubiquitous BBD cyanobacterium has been referred to in the literature based on sequence data of uncultured clones, as well as proposed members of existing or new cyanobacterial species [12,13,14,15,16,17], summarized in Casamatta *et al.* 2012 [18]. Sequence data also revealed that in Caribbean BBD several additional cyanobacterial genera may be present, in particular *Geitlerinema* and *Leptolyngbya* [19], and that BBD on the Great Barrier Reef develops from cyanobacterial “patches” dominated by *Blennothrix* [20]. Within these reports it became apparent that Pacific and Caribbean BBD cyanobacteria, each having near identical (97%–99% homology) 16S rRNA gene sequences, were substantively dissimilar. In terms of morphology, for example, whereas the Caribbean BBD cyanobacterium is narrow (*ca.* 4 μm wide), has smooth trichomes, and always possesses one rounded and one pointed tip on each filament (Figure 2), the BBD cyanobacterium from reefs of Palau is *ca.* 10 μm wide, has rounded tips at both ends, and has obvious constrictions between cells [14]. The Red Sea BBD cyanobacterium has blunt ends, and unlike the Pacific and Caribbean forms, which both have phycoerythrin, it possesses phycocyanin as the major light harvesting pigment [17]. Whereas the dominant Caribbean BBD cyanobacterium (and all other cyanobacteria isolated from Caribbean BBD) produces the cyanotoxin microcystin [21,22] BBD cyanobacteria from the Great Barrier Reef do not [23]. Thus, we are in agreement with the recent proposal [24] that there are phenotypically distinct strains of the dominant BBD cyanobacterium that possess near identical 16S rRNA gene sequences, comparable to those found in vibrios [25,26]. Casamatta *et al.* [18] used the polyphasic approach to characterize the Caribbean strain of the dominant BBD cyanobacterium under the International Code of Botanical Nomenclature (ICBN) as the new genus and species *Roseofilum reptotaenium*. This strain is the subject of this paper.

### 1.2. The Ecology of Caribbean Black Band Disease

The ecology of black band disease on Caribbean coral reefs has been studied for many years and this accumulation of information serves as an important background for understanding the ecology and role of *R. reptotaenium* in BBD pathobiology. Early studies [27] used oxygen- and sulfide-sensitive microelectrodes to investigate chemical microenvironments within the 1 mm thick BBD mat. Exactly analogous to cyanobacterial mats in sulfide-rich hot spring outflows, the BBD mat has steep vertical gradients of both oxygen and sulfide that overlap to form an oxygen/sulfide interface. This interface migrates vertically within the horizontally migrating BBD mat and is controlled by light presence/absence and intensity as it affects BBD cyanobacterial oxygenic photosynthesis [27] and by production of sulfide by the population of BBD sulfate reducing bacteria (SRB) [28,29]. These findings led to experiments on the ability of BBD cyanobacteria to tolerate sulfide, and it was shown that BBD cyanobacteria can perform sulfide-insensitive oxygenic photosynthesis [30]. The effect of sulfide was also investigated in terms of the coral host, and it was shown that exposure of healthy coral fragments to sulfide is toxic to the coral animal, leading to tissue lysis and death [31,32]. In several studies, it was shown that *R. reptotaenium* (as well as all other isolates of Caribbean BBD cyanobacteria tested) also produce toxins, specifically several variants of the cyanotoxin microcystin [21,22], but not saxitoxin or anatoxin-a [21]. The most common microcystin (MC) detected was MC-LR, the most toxic variant and the variant produced by *R. reptotaenium* [33]. Further studies revealed that, similar to sulfide, exposure of coral fragments to microcystin resulted in coral tissue lysis and death, and that sulfide and microcystin toxicity is synergistic [32]. An additional potential role of microcystin in BBD etiology was based on observations, using Scanning Electron Microcopy (SEM), that exposure of healthy coral fragments to 1 μM MC-LR elicited an increase in the number of heterotrophic bacteria on the coral surface, whereas exposure to higher MC-LR levels (50 μM and 100 μM) led to decreased numbers [33]. This finding is of interest since the BBD microbial community has a highly diverse population of BBD heterotrophs, of unknown function, that may be recruited from the surface mucopolysaccharide layer (SML) of the coral host [15,34]; thus it is possible the BBD cyanobacteria play a role in such recruitment. MC-LR was also shown [33] to promote or inhibit the growth of isolates of BBD heterotrophic bacteria, further suggesting that microcystin plays a role in structuring the BBD microbial community. Additionally, in a study investigating the anti-microbial properties of BBD and other coral reef cyanobacteria [35], we demonstrated that many of these cyanobacteria, including those isolated from BBD, produce bioactive compounds that suppress the growth of coral-associated bacteria.

As mentioned above, BBD is easily transmissible [7,8,36]. In a series of experiments investigating the roles of microcystin and sulfide in BBD toxicity we found that exposure of a BBD inoculum (full community) to sodium molybdate, a specific inhibitor of sulfate reduction, prior to inoculation of apparently healthy coral fragments prevented infection; yet exposure to sodium molybdate after coral fragments were successfully infected did not affect continued disease progress [33]. These experiments were repeated using laboratory cultures of *R. reptotaenium* with the same result [37]. Therefore, within this polymicrobial disease sulfide is required as an early step for infection, and the sulfate reducing bacteria in BBD can also be considered as BBD pathogens [37].

All of the studies discussed above indicate that BBD is complex and multifactorial. In this paper, we present additional new information about the biology of *Roseofilum reptotaenium* to further define the role of this BBD cyanobacterium, and the cyanotoxin that it produces, in the polymicrobial BBD of corals.

## 2. Methods

### 2.1. Isolation of Roseofilum reptotaenium Strains

*Roseofilum reptotaenium* strains 101-1 and 100-1 were isolated from BBD samples collected on a reef site in Frederiksted, St. Croix, U.S. Virgin Islands from two coral species, *Diploria strigosa* and *Siderastrea siderea*, as described in Casamatta *et al.* 2012 [18]. Laboratory cultures are maintained on ASNIII and marine BG11 at room temperature under natural light (by a window). One axenic strain was attained after repeated subculturing of individual filaments, and testing for contaminants on marine agar plates, however this strain lost viability after two weeks. The two isolates that maintained viability contain small heterotrophic bacteria closely associated with the extracellular polysaccharide sheath. A type strain of *R. reptotaenium* was deposited to the Provasoli-Guillard National Center for Marine Algae and Microbiota housed at the Bigelow Laboratory for Ocean Sciences and is available to investigators as culture CCMP_3313_.

### 2.2. Infection of Coral Fragments

*R. reptotaenium* strain 101-1 was used to inoculate coral fragments of the species *Diploria strigosa* collected from the Florida Keys National Marine Sanctuary (FKNMS) Key West Coral Nursery (permit number FKNMS-2007-026). After recovery from collection and acclimation in the laboratory, fragments were placed in 10 L aquaria with artificial seawater (ASW). A second aquarium served as a control (no inoculation of fragments). Aquaria were placed under a 12:12 h light:dark cycle with cool-white fluorescent light (Philips, F34T12/CW/RS/EW) at an intensity of 33 μE·m^−2^·s^−1^, measured using a Biospherical Instruments Quantum Scalar Irradiance meter (model QSL100), and at a temperature of 28 °C.

For inoculation, a small clump of *R. reptotaenium* biomass (*ca.* 5 mm in diameter) was gently placed on the surface of individual coral fragments with sterile forceps. The inoculum was held in place by a sterile Pasteur pipette resting on top of the inoculum until filaments were observed to adhere to the coral (1–2 day). Three experimental and three control fragments were used and the experiment repeated three times.

### 2.3. Scanning Electron Microscopy

Infected coral fragments, and also cultured *R. reptotaenium* (clumps), were fixed in 2.5% glutaraldehyde and maintained at 4 °C until processing. After fixation, fragments were dehydrated through a graded series of alcohol by placing them for 10 min in ethanol at concentrations of 20, 40, 60, 70, 90 (one wash each) and 100% (three washes). Fragments were then freeze fractured in liquid nitrogen and dried in a critical point dryer (Tousimis, Rockville, MD, USA), after which they were affixed to an aluminum stub using carbon adhesive tape and coated with palladium/gold using a sputter coater (SPI-Module). Fragments were viewed using a JOEL JSM-5900 LV Scanning Electron Microscope (JOEL USA, Peabody, MA, USA) at Florida International University.

### 2.4. Motility Patterns

Several aspects of motility patterns of *R. reptotaenium* were investigated, including chemotaxis to microcystin and sulfide, the effect of light, dark, and pH on clumping behavior, and observation and documentation of filament behavior in freshly collected black band disease and in culture.

Tactic responses were investigated using horizontal gradients of sulfide and microcystin, and directional *vs.* non-directional light sources, on ASN-III agar plates. To test for chemotactic response to sulfide, 25 mg of sodium sulfide nonahydrate (Na_2_S·9H_2_O) crystals were placed four cm from the spot where cyanobacterial filaments were inoculated. For inoculation, biomass (clumps of filaments approximately 5 × 5 mm) from stock cultures of both *R. reptotaenium* strains were placed on the agar surface using sterile forceps. In each of three (repeated) experiments, test plates (three replicate plates with sulfide plus two replicate control plates) were incubated on a 12:12 h light:dark cycle with cool-white fluorescent light at 30 μE·m^−1^·s^−1^ in a 28 °C incubator. Plates were oriented so that the side of the plate containing sulfide was closest to the light source. Control plates contained only cyanobacterial filaments and no sulfide. Tactic responses of filaments were documented in terms of their position on the plate as observed using a dissecting microscope. Migration of the filaments was marked, measured with a metric ruler, and photographed. Total migration was reported as the distance between the point where the sulfide crystals were plated and the closest cyanobacterial filament. As sulfide diffused into the agar, oxidation to polysulfide was observable as an opaque ring around the sulfide source. Sulfide concentrations within this gradient were measured using a modified Pachmayr Assay (described in Richardson and Castenholz, 1987) [38] which revealed that after 12 h the concentration of sulfide within the center of the sulfide-ring was as high as 12 mM. After 48 h of plating, sulfide was present at concentrations up to 1.5 mM.

For microcystin chemotaxis experiments, purified microcystin-LR (MC-LR), obtained from Kelly Rein at Florida International University, was used. For each assay a hole (challenge well) was punched into agar plates (one per plate) and filled with 50 µL of 1 µg/L, 50 µg/L, or 100 µg/L MC-LR from stock solutions made with artificial seawater (ASW). For the control plates, the challenge wells were created but not filled. Since microcystin is colorless, making visualization of MC diffusion impossible, 25 µL of safranin dye was mixed with 25 µL of 100 µg/L MC stock solution and pipetted into a challenge well. This mixture diffused in to the agar and formed a ring approximately 4 cm in diameter. Based on this, it was assumed that the microcystin diffused to the same extent as the dye. During the chemotaxis experiments only microcystin was used (no safranin). As in the sulfide chemotaxis assays, two control plates were used per trial with control plates containing only cyanobacterial filaments. Also as in the sulfide experiment, triplicate plates at each MC concentration were used, and the experiment was repeated three times. Assay plates were incubated as above. Plates were oriented so that the challenge well was closest to the light source. Tactic responses of filaments were observed as above in the sulfide assays.

During these experiments it was noted that in all control plates *R. reptotaenium* filaments moved to the light source, documenting positive phototaxis. To ensure responses to MC were not solely due to the positive phototactic response, the experiments were repeated utilizing a diffuse overhead light source. After inoculation the plates were incubated on a bench top within the laboratory at room temperature. The light intensity from the overhead fluorescent lights (33 μE·m^−1^·s^−1^) was comparable to the incubator light intensity. Tactic responses were observed and recorded as above.

Clumping behavior was investigated for both *Roseofilum* strains with experiments were performed in the light and the dark. Each BBD cyanobacterial strain was first homogenized by a hand-held homogenizer for 10 sec to disperse and suspend filaments. Homogenized biomass (500 μL) of each strain was dispensed into two 48-well plates (in triplicate for each plate), with one set of plates exposed to light (30 μE·m^−1^·s^−1^) and the other kept in the dark. Dark conditions were obtained by wrapping the plate in aluminum foil. The average biomass (dry weight) used per well for this experiment was 8.8 mg. The extent of filament clumping was recorded by taking digital pictures after 5, 10, 15 and 30 min. The rate of clumping was determined by measuring the diameter of biomass in each well using image analysis software (Image J software 1.40 g) and was expressed as percentage of area decrease relative to the freshly dispersed cultures. Documentation of overall behavioral patterns was conducted using a light microscope and direct observation of growth in 125 mL Erlenmeyer flasks.

### 2.5. Growth and Clumping as a Factor of pH

Both *Roseofilum* strains were investigated for their tolerance of, and ability to grow at, a range of pH values as well as for the effect of pH on clumping behavior. ASNIII medium was buffered with addition of MES buffer at a concentration of 0.5 g/L and adjusted by adding base (NaOH) or acid (HCl) to produce pH levels of 4.5, 5.5, 6.0, 7.0, 8.0, 9.0, and 10.0. Erlenmeyer flasks containing 30 mL of medium were inoculated with equal volumes of cultures (suspensions of filaments produced by homogenizing cultures with a hand-held mixer) in triplicate. Cultures were incubated at 27 °C. Biomass yield was determined as chlorophyll *a* concentration by filtering the whole culture volume (30 mL) through Whatman GF/B filters and extracting the biomass with 100% methanol at 4 °C for 24 h. Chlorophyll *a* concentration was determined by measuring absorbance at two wavelengths (653 nm and 666 nm) using a microplate reader (Synergy 2, BioTek Instruments, Inc., Winooski, VT, USA) and calculating chlorophyll *a* concentration according to Dere *et al.* (1998) [39]. The significance between growth at different pH values was tested by one way ANOVA and Bonferroni multiple-comparison.

### 2.6. Microcystin Exposure and Metabolite Production

A field study was conducted at the CARMABI Field Station in Curaçao to investigate the effect of microcystin (MC-LR) exposure on metabolite production by the black band community. Black band was collected, while SCUBA diving, from three BBD-infected colonies of *Diploria strigosa* using sterile needle-less 60 mL syringes. Water temperature on the reef was 27.5 °C and water depth was 4.0 m. Once onshore the BBD mat samples were concentrated by combining samples after allowing for clump formation. Homogenization of BBD mat (cyanobacterial filaments and the associated BBD microbial community) was performed by repeatedly pumping the combined samples through a needleless syringe. Sterile seawater was then added to increase the volume of dispersed BBD sufficient to inoculate the experiment. Exposure to MC-LR was carried out using 5 mL of dispersed sample in triplicate 15 mL Falcon tubes, at a final MC-LR concentration of 50 μg/L, for 24 h. Tubes were incubated in test tube racks in shaded natural light at ambient reef water temperature (28 °C) by standing in aquaria equipped with flow-through seawater. After a 24 h incubation with or without MC-LR the BBD communities in each tube were collected by vacuum filtration onto nylon filters, which were immediately folded, placed in cryogenic vials, and flash frozen at −78 °C. The frozen samples were then stored at −80 °C until the samples were extracted for metabolomics analysis.

### 2.7. Metabolite Extraction and Metabolomic Analysis Using UPLC-HRMS

Frozen filters from the field studywere placed in an empty petri dish maintained on a bed of ice. A cold (−20 °C) 1.3 mL aliquot of extraction solvent (40:40:20 acetonitrile:methanol:water containing 0.1 M formic acid) was added to thaw the sample and to extract the metabolome. The filter was then unfolded, placed sample side down in the extraction solvent, and allowed to extract at −20 °C for 20 min. The remainder of the extraction then proceeded as described previously [40,41]. The metabolomes for each sample were then measured via ultra-performance liquid chromatography—high resolution mass spectrometry (UPLC-HRMS) on an Exactive Plus Orbitrap MS (Thermo Fisher, Bremen, Germany) fitted with an UltiMate 3000 UPLC (Dionex, Sunnyvale, CA, USA) using described methods [41].

### 2.8. Metabolomic Data Processing and Analysis

Instrumental data files were generated by Xcalibur (Thermo Fisher) in .RAW format and were converted to .mzML format [42] using the msconvert software from ProteoWizard [43]. The open-source software, Maven (AKA mzroll) [44,45] was used to manually pick metabolite peaks based on known retention times and *m*/*z* values using the following criteria: exact mass within ±20 ppm (hard cut off), retention time ±4 min (soft cut off). Peak areas were exported to an Excel sheet as a .csv file. The data were then input into a metabolomics analysis program, MetaboAnalyst [46,47], and filtered using the interquantile range (IRN). The filtered data were normalized to a reference data set created from a pooled average sample selected from the control group. Fold changes and p values were then calculated from these normalized values.

## 3. Results

### 3.1. Laboratory Infection Using R. reptotaenium

**Figure 3:**
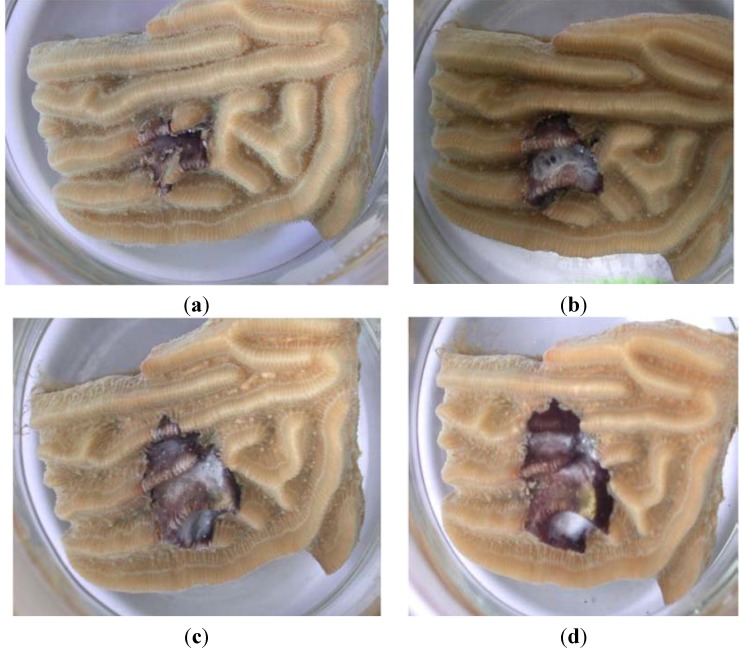
Infection of a fragment of *Diploria strigosa* using *R. reptotaenium* strain 101-1 under controlled laboratory conditions. Lesion progression over time is as follows: (**a**) t (time) = 7 d (days); (**b**) t = 14 d; (**c**) t = 21 d; (**d**) t = 28 d. Note the development of a white population of sulfide oxidizing bacteria (*Beggiatoa sp.*) on the mat (BBD) surface as the disease progresses.

Inoculation of all experimental fragments of *Diploria strigosa* using the laboratory culture of *Roseofilum* *reptotaenium* strain 101.1 resulted in BBD (representative fragment shown in Figure 3). All control (uninoculated) coral fragments remained healthy (not shown). Infection occurred after initial attachment of filaments to the coral surface, followed by an increase in cyanobacterial biomass to form a lesion (cyanobacterial mat) on the fragment surface. The lesion than increased in size (diameter) over the course of the experiment while forming a ring typical of naturally occurring BBD. As the ring (band) progressed to the edges of the coral fragments coral tissue was lysed exposing the underlying coral skeleton (Plates C and D of Figure 3). These results are in agreement with those of use of *R. reptotaenium* strain 100.1 to infect fragments of the host coral *Siderastrea siderea* [18].

### 3.2. Microscopic Analysis

**Figure 4:**
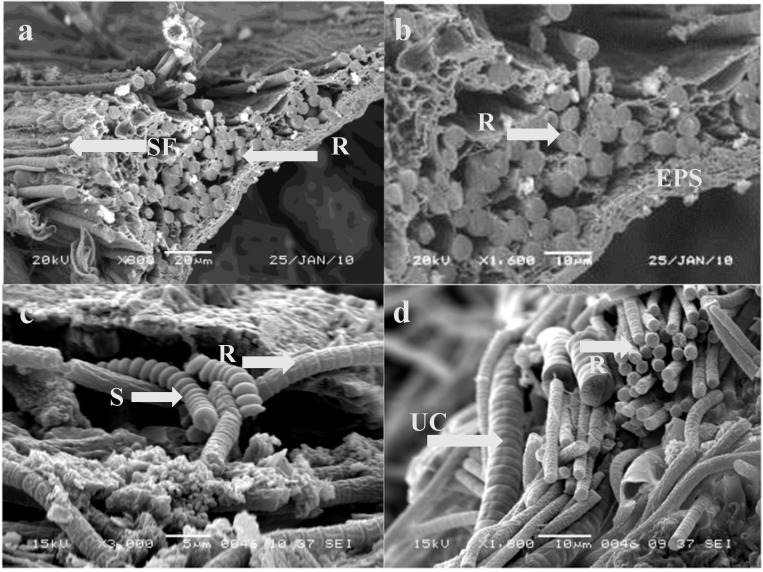
SEM of a freeze fractured migrating BBD mat on a fragment of *Siderastrea siderea* three weeks after laboratory infection with *Roseofilum* strain 100-1. (**a**) Well defined layer of *Roseofilum* filaments oriented parallel to one another; (**b**) Extracellular polysaccharide and smaller filaments associated with the matrix of *Roseofilum* filaments; (**c**) Filaments of *Spirulina* associated with *Roseofilum*; (**d**) Filaments of a large unidentified cyanobacterium. Note different scale bars in Plates. EPS = extracellular polysaccharide; S = *Spirulina*; R = *Roseofilum*; SF = small filaments; UC = unidentified large cyanobacterium.

Use of light microscopy and SEM to examine BBD from experimentally infected coral fragments revealed, in addition to *Roseofilum*, the presence of other morphologically distinct filamentous microorganisms (Figure 4). The most conspicuous were filaments identified as the cyanobacteria *Spirulina* and an unidentified cyanobacterium with filaments 8 μm in diameter. The cyanobacterial matrix consisted of a dense mat of filaments oriented parallel to one another (Figure 4a,b). These filaments were closely associated with other filamentous microorganisms (Figure 4c,d), revealing that a complex bacterial community developed within the experimentally induced BBD. Also of interest are small filaments of presumably heterotrophic bacteria in the matrix (Figure 4a). The identity and functional role of these members of the BBD community are not known, although heterotrophic bacteria have previously been seen in BBD using SEM [32].

### 3.3. Chemotactic Responses to Sulfide and Microcystin

Within hours following inoculation of *R. reptotaenium* onto test plates of ASN-III agar, the sodium sulfide crystals had melted and diffused into the agar, forming a visible ring of oxidized and precipitated polysulfide. The orientation of the plates, with the sodium sulfide placed between inoculated filaments and the light source, forced the positively phototactic filaments towards the sulfide ring. In all trials, *R. reptotaenium* filaments moved around the sulfide ring as they moved to the light source. Upon reaching the outer edge of the sulfide ring, the filaments either became immobile or migrated around the outer perimeter of the ring. The few filaments that entered the edge of the ring appeared to die. This assessment was based on their change in color from red to green, with the water soluble red pigment phycoerythrin released and leaving behind membrane bound chlorophyll *a*. There were no trials in which filaments entered and moved through the sulfide ring (Table 1). Filaments in control plates (no sulfide) travelled significantly farther (*p* < 0.05) to the light source than filaments with sulfide on that side of the plate. Filaments of *R. reptotaenium* plated onto ASN-III agar with a point source of microcystin revealed movement toward the challenge wells, and, in some trials, the filaments travelled directly to the edge of the challenge well. However, since the wells were between the inoculum and the light source this response could be due to positive phototaxis. To assess if this response was movement up the MC gradient or to the light source, plates were incubated under overhead (diffuse) light. Results showed that filaments did not travel in any specific direction, most likely due to the lack of a directional light source. There was no statistical difference (*p* = 0.75) in distance travelled by filaments on control and experimental plates (Table 1). Therefore, the significant movement in the experiments with the directional light source was the result of phototaxis.

**Table 1:** Chemotactic responses of *Roseofilum reptotaenium* to gradients of sulfide and microcystin-LR on ASN-III agar plates in directional and diffuse light. Data are presented as the final average distance, with ranges, of filaments from the sulfide or microcystin source. Nine plates were used for each trial. *p* values are for test substances in comparison to the controls.

Test Substance and Light Source	Distance from Sulfide or Microcystin Source (cm)	*p* Value
Directional light		
Sulfide	3.1 ± 1.7	
Control	1.8 ± 3.1	<0.05
MC-LR (1 µg/L)	1.2 ± 1.1	
MC-LR (50 µg/L)	1.1 ± 1.1	
MC-LR (100 µg/L)	1.2 ± 1.7	
Control (MC-LR)	1.3 ± 1.3	0.99
Diffuse light		
MC-LR (1 µg/L)	3.5 ± 1.0	
MC-LR (50 µg/L)	3.5 ± 0.5	
MC-LR (100 µg/L)	3.6 ± 0.3	
Control	3.6 ± 0.5	0.75

### 3.4. Clumping Behavior

**Figure 5:**
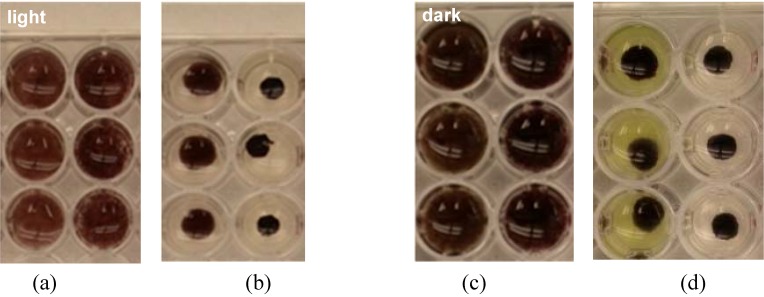
Clumping behavior of *Roseofilum reptotaenium* in the light (**a**,**b**) and dark (**c**,**d**). Filaments of strains 101-1 and 100-1 (left and right wells in each panel, respectively) were dispersed by homogenization and photographed over time. Panels a and c: *t* = 0; panels b and d: *t* = 30 min.

Conspicuous clumping behavior was observed by both *Roseofilum* strains in liquid culture. Since this behavior is potentially important in BBD mat formation and pathobiology, clumping was investigated under both light and dark conditions (Figure 5). The most pronounced clumping was observed for the *Roseofilum* strains in the light, with dispersed filaments contracting to an area that was reduced by 85% (strain 100-1) and 73% (strain 101-1). In the dark, *Roseofilum* filaments contracted to an area that was 78% and 69%, respectively, of the dispersed area. Thus, for each strain the rate of clumping was faster in the light than in the dark, and strain 100-1 clumped faster than 101-1. In all cases, clumping rate was most rapid during the first five minutes, and then continued at decreased rates.

**Figure 6:**
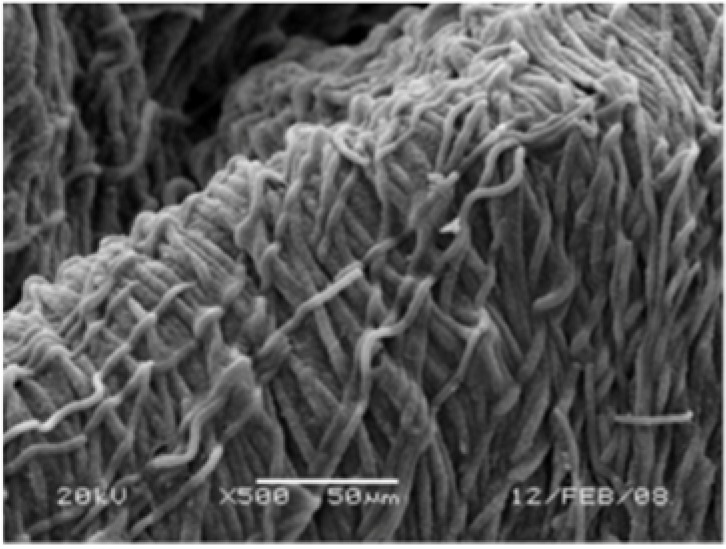
SEM of cross-section of a clump formed by *Roseofilum* strain 101-1 after dispersal and clump formation in wells. Filaments are arranged in a tight and regular pattern.

SEM analysis of the clumps formed by *Roseofilum* in the wells (shown in Figure 5) revealed a unidirectional orientation of filaments (Figure 6) similar to that observed in experimentally induced BBD lesions and natural samples of BBD [32].

**Figure 7:**
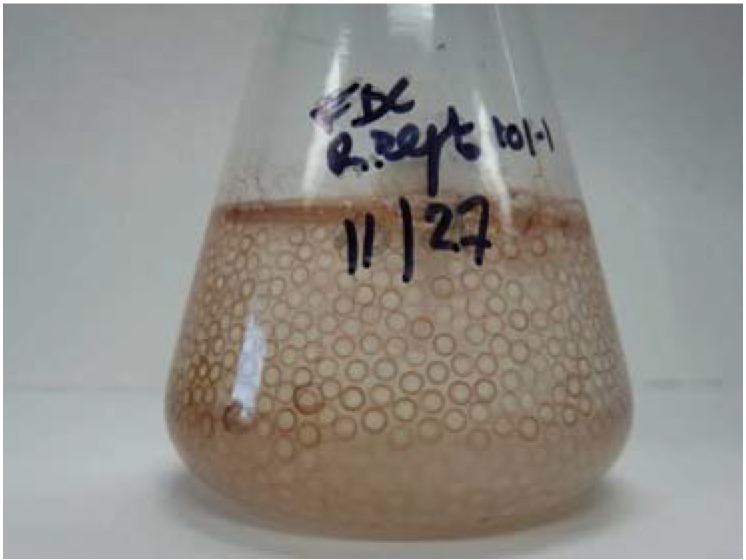
Ring formation of *R. reptotaenium* grown in liquid culture in ASN III. Filaments routinely form this pattern on the sides of Erlenmeyer flasks. Photograph by Florian De Clercq.

In contrast to the formation of clumps after filament dispersal and suspension, cultures grown undisturbed in liquid media in Erlenmeyer flasks formed striking, and possibly unique, patterns of rings and circles (Figure 7). These rings are consistently of uniform size and are approximately 3 mm in diameter (outer edge).

### 3.5. Effect of pH on Growth and Clumping

**Figure 8:**
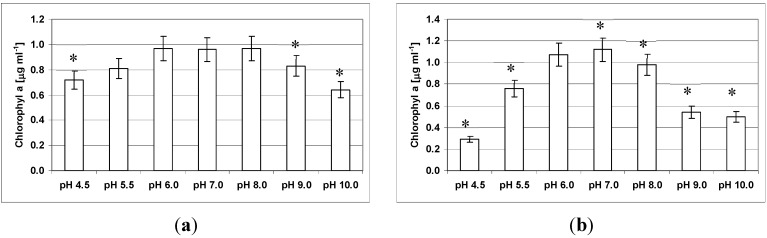
Effect of different pH values on growth of *Roseofilum* strains 100-1 (**a**) and 101-1 (**b**). Statistical significance, when compared with biomass yield at pH 8.0 for each strain, is indicated by *.

Both *Roseofilum* strains were capable of growth at a wide range of pH values (Figure 8), with strain 100-1 more pH tolerant than strain 101-1. For strain 100-1 (Figure 8a) the biomass yield was not significantly different from pH 8.0 at values of 5.5, 6.0 and 7.0. Significant decreases in biomass yield were recorded at pH values 4.5, 9.0 and 10.0 (*p* < 0.05). Strain 101-1 exhibited significantly different growth at all pHs in comparison to growth at pH 8.0 (*p* < 0.05), with the highest biomass yield at pH 7.0 (Figure 8b). All cultures remained viable across the range of pH values.

In addition to the differences in growth yield when pH was varied, there was a difference in growth characteristic. Both strains formed tightly packed clumps that floated at the surface at pH values of 4.5, 5.5, 6.0 and 10.0 for strain 100-1, and pH 4.5 and 5.5 for strain 101-1. At all other pH values, the filaments were observed to attach to and form a film over the sides of the flasks.

### 3.6. Effect of Microcystin on Metabolite Production in the BBD Community

**Figure 9:**
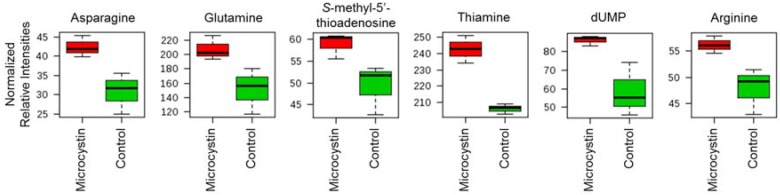
Box and whisker plots representing the range of normalized relative intensities for significant metabolic changes in the BBD microbial community after 24 h exposure to 50 µg/L MC-LR. Data are from UPLC-HRMS measurement of triplicate samples. Significance was determined by a fold change >1.5 and a *p* value of <0.05.

A total of 135 metabolites were detected in the metabolomics experiment. The most significant effect on the BBD microbial community after the 24 h exposure to 50 μg/L MC-LR was an increase (*p* ≤ 0.05 and fold change ≥1.5) in the pool (*i.e.*, relative concentration) of six metabolites (asparagine, glutamine, *S*-methyl-5’-thioadenosine, thiamine, dUMP and arginine) as compared to the control (Figure 9). Eight other metabolites exhibited potentially significant changes (*p* ≤ 0.1 and/or fold change ≥ 1.25) (Table 2). All significant responses were noted to be increases in the metabolite pools. The entire data set (135 metabolites) is presented in Supplemental Table 1.

**Table 2:** Changes in concentration, presented as fold values, for metabolites detected in the BBD community after 24 h exposure to 50 μg/L MC-LR as calculated from MetaboAnalyst 2.5 processed data [47]. Metabolites in the microcystin treated BBD community with the most robust differences (*p* ≤ 0.05 and fold change ≥1.5) from the control (*t* = 24, no exposure to microcystin) are bolded. Other potentially interesting metabolites with less robust changes (*p* ≤ 0.1 and/or fold change ≥1.25) are italicized.

Metabolite	Fold Change	*p* Value
*N,N-Dimethylglycine*	*1.299*	*0.012*
*Betaine*	*1.281*	*0.051*
*Thymine*	*1.703*	*0.082*
**l-Asparagine**	**2.486**	**0.020**
**l-Glutamine**	**2.392**	**0.044**
*l-Glutamate*	*1.378*	*0.005*
*Xanthine*	*1.388*	*0.103*
**l-Arginine**	**1.588**	**0.039**
N*-Carbamoyl-l-Aspartate*	*1.349*	*0.086*
*Deoxyuridine*	*2.015*	*0.056*
**Thiamine**	**1.630**	**0.018**
***S*-Methyl-5’-Thioadenosine**	**1.666**	**0.048**
N*-Acetyl-Glucosamine-1*/*6-Phosphate*	*1.423*	*0.019*
**dUMP**	**2.840**	**0.037**
*Aminoimidazole Carboxamide Ribonucleotide*	*1.471*	*0.001*
*CDP-Choline*	*1.426*	*0.078*
*UDP-N-Acetyl-glucosamine*	*1.376*	*0.034*
*NAD+*	*1.435*	*0.008*
*Cyclic bis-3,5-dimeric GMP*	*1.802*	*0.065*
*FAD*	*1.367*	*0.042*

## 4. Discussion

Based on previous work by our group and others (discussed in detail in the introduction), and our new results presented here, we now know that *R. reptotaenium* plays several different roles as a member of the pathogenic polymicrobial BBD of corals. These include initial adherence to the host coral, which allows infection; creation of an anoxic microenvironment that enriches for an additional BBD pathogen (SRB); toxin production; synergy with another toxic member (SRB) of the community; and an influence of the cyanotoxin MC-LR on the metabolism of the BBD microbial community.

The motility patterns of *R. reptotaenium* appear to be very important in the role of this cyanobacterium as a pathogen. The ability to adhere to and grow as a mat on the surface of a coral host together with the continual and directional gliding motility of *R. reptotaenium* results in the compact and discrete band, or fabric, of the disease. Band formation appears to be controlled by tactic responses to light and sulfide as well as by the effect of filament morphology on movement. *R. reptotaenium* exhibits a step-down photophobic response (L. Richardson, personal observation), which would tend to cue the filaments to move towards the band surface. Similarly, negative taxis to sulfide (Table 1) would keep filaments away from the sulfide-rich deeper layers of the BBD mat. Clumping behavior in the light and dark (Figure 5) would enhance the maintenance of the band at the interface between healthy coral tissue and exposed coral skeleton, further enhanced by a clumping response to coral mucus, which is slightly acidic [48]. BBD mat formation may also be based on the circular motility that produces rings on the sides of flasks in liquid culture (Figure 7), a pattern that may be due to a preferred gliding motility in which the pointed end of the filament often serves as the leading end. This characteristic is notable when observing freshly collected BBD under the microscope, when it can be seen that individual filaments of *R. reptotaenium* move out of clumps of BBD biomass with the tapered end serving as the leading tip (L. Richardson, personal observation). It would be interesting to model the effect of such directional gliding motility (positive phototaxis, step-up photophobic response and negative sulfide chemotaxis), coupled with the effect of traveling in a circle when on a surface due to the presence of one tapered leading end, using the motility model presented by Tamulonis and Kaandorp [49] and see if the model would result in the formation of 3 mm rings.

The results of the mat forming behavior of *R. reptotaenium* is also visible in the SEMs of BBD lesions produced in the laboratory (Figure 3) and inside of clumps of cultures of *R. reptotaenium* (Figure 6). The fine structure revealed in both of these closely resembles SEMs produced using the mat of freshly collected BBD [50]. In these prior investigations it was also revealed that at the leading edge of the BBD mat *R. reptotaenium* tunnels through coral tissue, and under the mat itself filaments of *R. reptotaenium* can be seen boring into coral skeleton [51]. The cues for such activity are not known.

We have shown, here and in our previous work, that unilalgal cultures of *R. reptotaenium* can initiate BBD on healthy coral fragments under controlled conditions in the laboratory (Figure 3). Close observation of the infection process shows that the cyanobacterium carries out the first step in the infection process—adhesion to the surface of the coral. Once adhered, an increase in cyanobacterial biomass occurs as the population grows and accumulates. During this time, anaerobic zones are formed within the developing mat which then enrich for sulfate reducing bacteria, demonstrated over time using molecular techniques during *in situ* BBD development on the Great Barrier Reef [52] and by observation of the appearance of sulfide oxidizing bacteria on the band surface (Figure 3). As sulfide accumulates, an active sulfur biogeochemical cycle is brought into play, as evidenced by sulfide microprofiles in the band [27]. As discussed above, the SRB population in BBD, along with the BBD cyanobacteria, is required for BBD pathogenicity [37].

We know that both the cyanobacterial and SRB pathogens are producing toxic compounds (microcystin and sulfide) that act synergistically to kill coral tissue. As the animal tissue lyses and organic carbon is released, a highly variable heterotrophic bacterial community develops. The role of this community in BBD pathobiology is not known, but it is known that populations of BBD heterotrophic bacteria are highly diverse. A meta-analysis [53] was carried out that analyzed 10 published clone libraries [12,13,14,15,16,19,20,28,29,53] produced from 87 BBD samples, collected from 16 species of scleractinian corals in 10 different geographic locations. This study revealed 326 unique operational taxonomic units (OTUs) in the BBD clone libraries that corresponded to heterotrophic bacteria, with representatives from nine phyla, including five subdivisions within the most diverse phylum (the Proteobacteria). Three OTUs, (one Cytophaga–Flavobacter–Bacteriodetes and two Alphaproteobacteria), were detected in 13% of the samples. The remaining 323 unique OTUs were present in <10% of the samples and 73% of these were singletons [54]. Overall, the Alphaproteobacteria were the most diversely represented group. While the functional roles of these BBD heterotrophs are not yet known, experiments are underway. We have recently detected production of quorum sensing signal molecules by BBD bacterial isolates [55], and the next step will be to determine what genes are being regulated by these autoinducers.

In addition to a potential role of quorum sensing in BBD, the production of microcystin by *R. reptotaenium* may be influencing the community of BBD heterotrophic bacteria. Besides stimulating/inhibiting bacterial growth on the surface of healthy corals and growth of coral-associated bacterial isolates [33], we have shown here that exposure to MC-LR directly alters the pools of a number of physiologically important metabolites (Figure 9 and Table 2). In particular, increased production of asparagine, glutamine, and *S*-methyl-5’-thioadenosine is important for synthesis of both DNA and RNA. These are all precursors to the synthesis of the purine ring [56] needed for nucleic acid monomers. Thiamine and dUMP, also shown to increase with microcystin exposure (Figure 9), are also intimately linked to nucleic acid biosynthesis and are part of the pyrimidine pathway. As metabolites are intimately linked through metabolic pathways, alterations in metabolite pools that do not alone meet stringent criteria for significance can still be used to corroborate changes in significant metabolites if they cluster within organized biosynthetic units. Two other purine linked (xanthine and aminoimdiazole carboxamide ribonucleotide) and four pyrimidine linked (UDP, UPD-*N*-acetyl-glucosamine, thymine, and cyclic bis-3,5-dimeric GMP) showed pool size alterations nearing significance as judged by the most stringent criteria. Further, the cofactors NAD^+^ and FAD were altered, although the magnitude of the fold change was not as robust as for other metabolites. Thus, based on the data presented here, microcystin may affect the metabolism of the BBD community by altering nucleic acid biosynthesis.

## 5. Conclusions

In summary, *Roseofilum reptotaenium* is, to our knowledge, the first known cyanobacterium that is an active member of a pathogenic polymicrobial consortium. It plays several roles in a globally distributed coral disease that is recognized as contributing to reef destruction world-wide [4]. While this paper has focused on the Caribbean strain of the dominant BBD cyanobacterium, we recognize that the Indo-Pacific and Red Sea strains (designated as an *Oscillatoria* spp. [20] and *Pseudoscillatoria coralli* [17], respectively), may have different mechanisms of pathogenicity and contributions to BBD etiology. Many elegant studies have been conducted describing BBD in these regions of the world [20,24,52,54], and it will be very interesting to see the results of laboratory studies investigating the ecophysiology of these strains for comparison to *R. reptotaenium*.

## Supplementary Materials

Supplementary materials can be accessed at: http://www.mdpi.com/2075-1729/4/4/968/S1.
